# Incidence change of postoperative delirium after implementation of processed electroencephalography monitoring during surgery: a retrospective evaluation study

**DOI:** 10.1186/s12871-023-02293-9

**Published:** 2023-10-04

**Authors:** Yi-Chen Chen, I-Yin Hung, Kuo-Chuan Hung, Ying-Jen Chang, Chin-Chen Chu, Jen-Yin Chen, Chung-Han Ho, Chia-Hung Yu

**Affiliations:** 1 Department of Medical Research, Chi Mei Medical Center, 901 Zhonghua Road, Yongkang District, Tainan, Taiwan; 2 Department of Anesthesiology, Chi Mei Medical Center, 901 Zhonghua Road, Yongkang District, Tainan, Taiwan; 3 Department of Hospital and Health Care Administration, College of Recreation and Health Management, Chia Nan University of Pharmacy and Science, 60 Erren Road, Rende District, Tainan, Taiwan; 4 Department of Recreation and Health Care Management, College of Recreation and Health Management, Chia Nan University of Pharmacy and Science, 60 Erren Road, Rende District, Tainan, Taiwan; 5 Department of Information Management, Southern Taiwan University of Science and Technology, 1 Nantai St, Yongkang District, Tainan, Taiwan; 6 Department of Computer Science and Information Engineering, Southern Taiwan University of Science and Technology, 1 Nantai St, Yongkang District, Tainan, Taiwan

**Keywords:** Postoperative delirium, Processed electroencephalography, General anesthesia, Intravenous patient-controlled analgesia

## Abstract

**Background:**

Postoperative delirium (POD) is a common complication in the elderly, which is associated with poor outcomes after surgery. Recognized as predisposing factors for POD, anesthetic exposure and burst suppression during general anesthesia can be minimized with intraoperative processed electroencephalography (pEEG) monitoring. In this study, we aimed to evaluate whether implementation of intraoperative pEEG-guided anesthesia is associated with incidence change of POD.

**Methods:**

In this retrospective evaluation study, we analyzed intravenous patient-controlled analgesia (IVPCA) dataset from 2013 to 2017. There were 7425 patients using IVPCA after a noncardiac procedure under general anesthesia. Patients incapable of operating the device independently, such as cognitive dysfunction or prolonged sedation, were declined and not involved in the dataset. After excluding patients who opted out within three days (N = 110) and those with missing data (N = 24), 7318 eligible participants were enrolled. Intraoperative pEEG has been implemented since July 2015. Participants having surgery after this time point had intraoperative pEEG applied before induction until full recovery. All related staff had been trained in the application of pEEG-guided anesthesia and the assessment of POD. Patients were screened twice daily for POD within 3 days after surgery by staff in the pain management team. In the first part of this study, we compared the incidence of POD and its trend from 2013 January–2015 July with 2015 July–2017 December. In the second part, we estimated odds ratios of risk factors for POD using multivariable logistic regression in case-control setting.

**Results:**

The incidence of POD decreased from 1.18 to 0.41% after the administration of intraoperative pEEG. For the age group ≧ 75 years, POD incidence decreased from 5.1 to 1.56%. Further analysis showed that patients with pEEG-guided anesthesia were associated with a lower odd of POD (aOR 0.33; 95% CI 0.18–0.60) than those without after adjusting for other covariates.

**Conclusions:**

Implementation of intraoperative pEEG was associated with a lower incidence of POD within 3 days after surgery, particularly in the elderly. Intraoperative pEEG might be reasonably considered as part of the strategy to prevent POD in the elder population.

**Trial registration:**

Not applicable.

**Supplementary Information:**

The online version contains supplementary material available at 10.1186/s12871-023-02293-9.

## Background

Delirium is a neurocognitive disorder characterized by acute disturbance in attention, awareness, and cognition with a fluctuating course [1]. Postoperative delirium (POD) is a common complication in the elderly that usually occurs within 1 to 3 days after surgery [2]. Its incidence has been reported to be 5–52% in the elderly after noncardiac surgery [1, 3]. POD is associated with delayed recovery, prolonged hospital stay, increased mortality, and high expenditure and burden on the healthcare system [4, 5]. Furthermore, patients with POD may be at a higher risk of long-term cognitive dysfunction [6]. Since there are currently no effective treatments, prevention of POD is paramount. In addition to common risk factors for incident delirium, such as dementia, old age, severe illness, immobility, electrolyte imbalance, and malnutrition [7], POD is also related to anesthetic dose and longer burst suppression on electroencephalogram [8–11].

Electroencephalogram is a common tool for brain monitoring. Processed electroencephalogram (pEEG) simplifies the interpretation. It reflects near real-time cerebral activity during general anesthesia, which makes it possible to individualize anesthetic dosages according to the patient’s sensitivity and cerebral activity [12–14]. Literature reported that the dosage of anesthetics could be reduced safely with intraoperative pEEG without increasing intraoperative awareness [15, 16]. In some meta-analyses, intraoperative pEEG monitoring was demonstrated to be useful in mitigating postoperative cognitive dysfunction and POD [12, 17]. However, challenged by the results of the ENGAGES randomized controlled trial in 2019, the effect of pEEG on reducing POD remains controversial [18]. This is further supported by another meta-analysis, which concluded insufficient evidence for the benefit of pEEG-guided anesthesia in reducing POD [19].

In this study, we performed a retrospective plausibility evaluation of the occurrence of POD before and after administering intraoperative pEEG monitoring. We aimed to evaluate whether the use of intraoperative pEEG is associated with a significant change in the incidence of POD.

## Methods

### Data

Data were retrieved from the postoperative intravenous patient-controlled analgesia (IVPCA) dataset of Chi Mei medical center from January 1, 2013, to December 31, 2017. This dataset was established for quality management to monitor side effects, including delirium. Chi Mei medical center is a tertiary health service unit and teaching hospital in southern Taiwan. Patients who were willing to use IVPCA for pain management after surgery were evaluated by the pain management group to ensure that their cognitive function was adequate to understand and operate the PCA pump correctly. Patients unable to operate the pump independently or who required prolonged sedation were not enrolled. Once the pump was set, the pain management team would visit the patient twice daily and follow up for 3 days. Records of 7425 eligible individuals were extracted from the dataset during this period. Patients who dropped out within 3 days or those without a complete hospitalization record were excluded (N = 110). Patients with missing detailed information were excluded (N = 24). Therefore, 7318 participants who used IVPCA after a noncardiac surgery under general anesthesia were enrolled in this study.

### Study design

We performed a retrospective plausibility evaluation to assess the change in the incidence of POD after intraoperative pEEG during general anesthesia administered in July 2015. A plausibility evaluation controls confounders by comparing post-intervention and baseline groups to examine the improvements in target indicators [20]. The incidence of POD is represented per 1000 persons and divided into four quarters of the year. We analyzed the quarterly incidence of POD for distinct periods before and after intraoperative pEEG administration to examine if there was a significant change. Before the introduction of pEEG, minimal alveolar concentration measured from end-tidal anesthetic had been used as a general guide for immobility and decreasing awareness [21]. Processed electroencephalogram was introduced in July 2015 to monitor anesthetic depth and applied to all patients undergoing surgery under general anesthesia. The monitor started before induction throughout the surgery until the patient fully awakened, and all the staff was educated to adjust anesthetics according to the value and clinical presentation.

### Measures of outcomes

In this study, the patients using an IVPCA set were followed up to 3 days to ensure adequate pain control and evaluated side effects, including POD occurrence during the postoperative period. POD was initially screened by nurses in the pain management team using the Nursing Delirium Screening Scale. Based on this scale, patients were assigned a score of 0 to 2 for each of the five symptoms: disorientation, inappropriate behavior, inappropriate communication, illusion/hallucination, and psychomotor retardation (0 = not present, 1 = mild, and 2 = severe) [22]. We used ≥ 1 as the threshold, which provided higher sensitivity (67%) and adequate specificity (93%) [23]. After the initial assessment, an anesthesiologist further evaluated patients with delirium scores ≥ 1 and suspected delirium episodes. The diagnosis of POD was confirmed according to the Confusion Assessment Method diagnostic algorithm [24].

#### Covariates

Intraoperative pEEG monitoring with either bispectral index or state entropy and response entropy has been launched since July 2015 in this medical center to optimize the consumption of anesthetics and minimize burst suppression under general anesthesia. It is recommended to maintain the index values between 40 and 60 during anesthesia and avoid burst suppression as much as possible. We evaluated the demographic characteristics of the participants, including age, sex, PCA formula, American Society of Anesthesiologists (ASA) classification, emergency, and surgery type, to compare the standard mean differences before and after intraoperative pEEG administration. Age is presented as a categorical variable with four groups: <45, 45–59, 60–74, and ≥ 75 years. IVPCA users were prescribed one of the five formulas: morphine alone, morphine plus ketorolac, fentanyl, fentanyl plus ketorolac, and morphine plus ketamine. The detailed regimen is provided in an additional table file [see Additional file 1]. ASA classification was assigned as recommended by the ASA physical status classification system according to the patient’s underlying condition [25]. For patients with severe or life-threatening disease processes, a classification ≥ 3 was assigned. Patients with a healthy or mild to moderate systemic disease were assigned a classification < 3.

### Statistical analysis

Given the sample sizes collected in the dataset and the incidences of POD reported in previous meta-analysis, the power to detect the difference in the primary outcome was calculated to be more than 90% on the basis of 2-sided ɑ<0.05 [19]. The occurrence of POD is presented as the frequency with percentage and compared using Pearson’s chi-square test between the periods before and after the implementation of intraoperative pEEG monitoring. The delirium score of patients within 72 h is presented as median with quartiles, and non-parametric testing was performed to examine the difference. Quarterly data of POD in PCA populations are shown as incidence per 1000 persons and the number of patients. Piecewise linear regression was used to estimate the mean and slope of the quarterly POD trend across the two periods. Subgroup analysis was performed to elucidate the incidence of POD stratified by four age groups: <45, 45–59, 60–74, and ≥ 75 years.

To analyze potential confounders for POD, patients with and without delirium were categorized into case and control groups in a case-control design. Participant information, including intraoperative pEEG monitoring, sex, the formula of PCA, ASA, and emergency, is presented as the frequency with percentage and age as mean with standard deviation. The differences in covariates between the case and control groups were examined using Pearson’s chi-square test and Fisher’s exact test for categorical variables, and Student’s t-test for age. Logistic regression analysis was used to estimate the risk factors for POD, including pEEG-guided anesthesia, age per 10 years, sex, PCA formula, ASA, emergency, and surgery type. Statistical analyses were conducted using SAS statistical software (version 9.4; SAS Institute, Inc., Cary, NC, USA) and StataCorp. 2017 (StataCorp LLC, Stata: Release 15. Statistical Software. College Station, TX, USA). Statistical significance was set at a *P*-value of < 0.05.

## Results

**Fig. 1:**
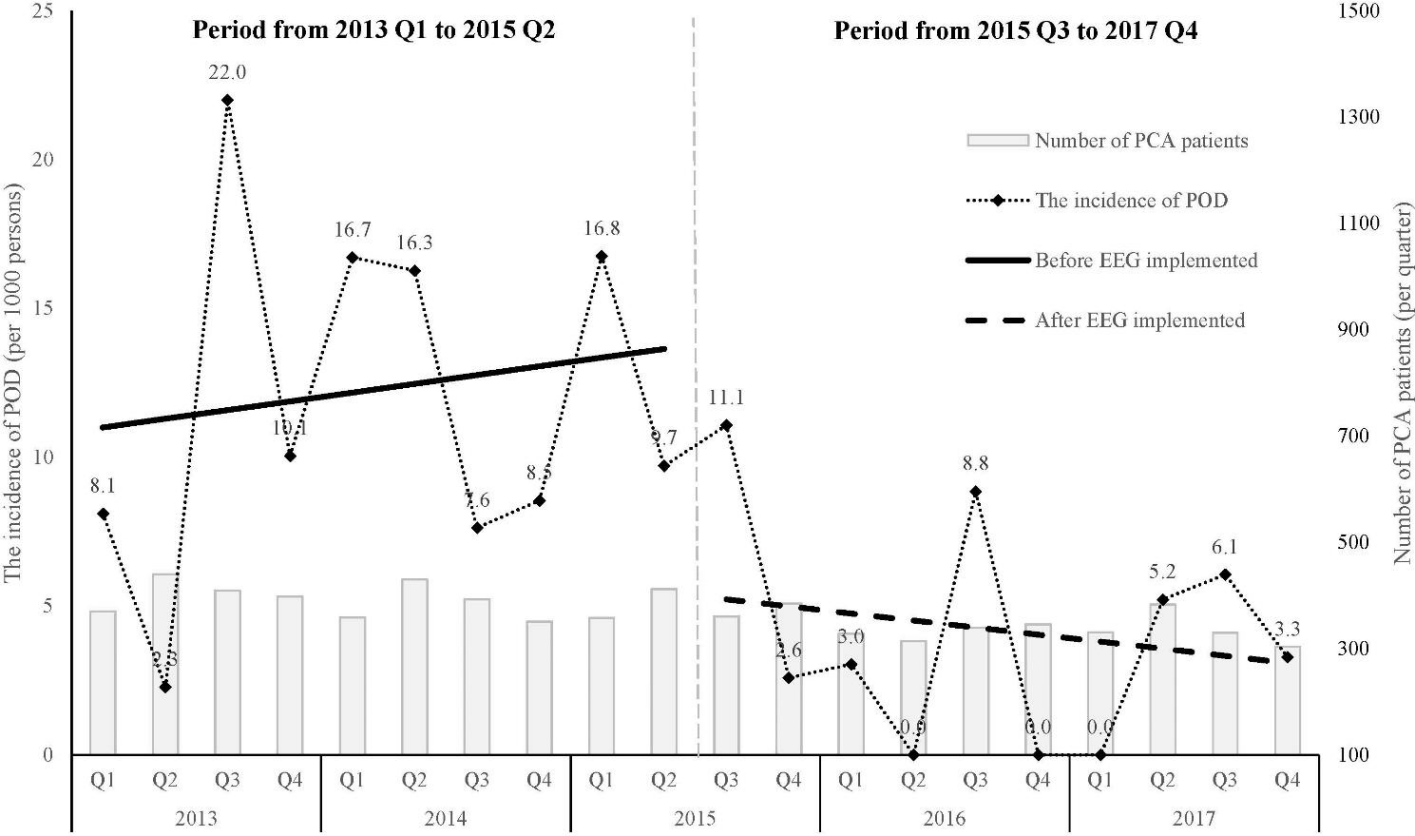
Quarterly incidences of POD and the trend before and after intraoperative pEEG implementation

From 2013 to 2017, 3907 and 3411 patients were enrolled before and after the implementation of intraoperative pEEG monitoring in July 2015, respectively. The incidence of POD and the number of patients using PCA by quarter between 2013 and 2017 are presented in Fig. 1. The mean rate of POD in a quarter was 13.64 and 5.46 per 1000 persons before and after the launch of pEEG-guided anesthesia, respectively. The rate of POD per quarter increased with a slope of 0.29 per 1000 persons per quarter before the use of pEEG and afterward decreased gradually with a slope of − 0.24 per 1000 persons per quarter. The incidence of POD and delirium score are shown in Table 1. The incidence of POD was significantly lower after administration of intraoperative pEEG than before (0.41% vs. 1.18%, *P* < 0.001), whereas the delirium scores remained similar (median, Q1–Q3: 1, 1–2 vs. 1, 1–2, *P* = 0.590). Baseline characteristics are presented in an additional table file [see Additional file 2]. The standardized mean differences in all characteristics were less than 20%.

**Table 1:** Incidence of POD in PCA users before and after intraoperative pEEG implemented

	Before EEG implementation N (%)	After EEG implementation N (%)	*P*-value
	3907 (53.39)	3411 (46.61)	
POD	46 (1.18)	14 (0.41)	< 0.001
Delirium score			
Median (Q1-Q3)	1 (1–2)	1 (1–2)	0.590

*P-value was derived from Pearson’s chi-square test for categorical variables and Wilcoxon rank sum test for delirium scores

POD, postoperative delirium; PCA, patient-controlled analgesia; pEEG, processed electroencephalography

To identify the risk factors for POD, patients were categorized into case and control groups in a case-control setting. Demographic characteristics of potential confounders in both groups are presented in Table 2. There were 7258 patients in the control group without POD and 60 in the case group. In the case groups, patients are found with a higher proportion having surgery without intraoperative pEEG (76.67% vs. 53.2%, P-value < 0.001), older age (P-value < 0.001), ASA ≧ 3 (88.33% vs. 60.57%, P-value < 0.001), and emergency surgery (15% vs. 5.14%, P-value = 0.004). There was also a difference in the formula used for PCA between the two groups (P-value = 0.002). These covariates were further analyzed using logistic regression to determine the effect of each covariate (Table 3). After adjustment for other covariates, patients with intraoperative pEEG monitoring during surgery were associated with lower odds of developing POD within 3 days after surgery than those without pEEG (adjusted odds ratio [aOR] 0.33, 95% confidence interval [CI] 0.18–0.60). In addition, after adjusting for other covariates, it was found that patients had higher odds of developing POD within 3 days if they were older (per 10 years, aOR 2.53, 95% CI 1.96–3.27) or underwent emergency surgery (aOR 3.59, 95% CI 1.73–7.47).

**Table 2:** Demographic characteristics of potential confounders for patients with or without POD

	POD, N (%)		*P*-value
	Control group 7258 (100)	Case group 60 (100)	
Intraoperative pEEG			
Yes	3397 (46.80)	14 (23.33)	< 0.001
No	3861 (53.20)	46 (76.67)	
Age, mean ± SD	57.07 ± 15.62	74.32 ± 10.80	< 0.001
<45	1550 (21.36)	1 (1.67)	< 0.001
45–59	2229 (30.71)	6 (10.00)	
60–74	2496 (34.39)	17 (28.33)	
≥75	983 (13.54)	36 (60.00)	
Sex			
Male	3299 (45.45)	31 (51.67)	0.336
Female	3959 (54.55)	29 (48.33)	
Formula			
I (morphine)	3901 (53.75)	48 (80.00)	0.002
II (morphine + keto)	3222 (44.39)	12 (20.00)	
III (fentanyl)	109 (1.50)	0 (0.00)	
IV (fentanyl + keto)	12 (0.17)	0 (0.00)	
V (morphine + ketamine)	14 (0.19)	0 (0.00)	
ASA classification			
<3	2862 (39.43)	7 (11.67)	< 0.001
≥3	4396 (60.57)	53 (88.33)	
Emergency	373 (5.14)	9 (15.00)	0.004

*P-value was derived from Pearson’s chi-square test and Fisher’s exact test, when the expected value was less than five, for categorical variables and Student’s t-test for continuous variables

POD, postoperative delirium; pEEG, processed electroencephalography; ASA, American Society of Anesthesiologists

**Table 3:** Risk factors for POD within 72 h after noncardiac surgery

Variable	Crude OR (95% CI)	Adjusted OR (95% CI)
Intraoperative pEEG		
Yes	0.35 (0.20–0.64)	0.33 (0.18–0.60)
No	Ref.	
Age, per 10 years	2.81 (2.19–3.60)	2.53 (1.96–3.27)
Sex, male	1.28 (0.77–2.12)	1.33 (0.80–2.20)
Formula		
I (morphine)	Ref.	Ref.
II (morphine + keto)	0.31 (0.17–0.58)	0.57 (0.30–1.07)
III (fentanyl)	0.37 (0.02–6.07)	0.39 (0.02–6.39)
IV (fentanyl + keto)	3.22 (0.17–61.85)	3.61 (0.14–94.84)
V (morphine + ketamine)	2.77 (0.15–52.05)	
ASA		
≥ 3	4.64 (2.16–9.99)	1.24 (0.56–2.73)
Emergency	3.40 (1.69–6.85)	3.59 (1.73–7.47)

POD, postoperative delirium; OR, odds ratio; CI, confidence interval; pEEG, processed electroencephalography; ASA, American Society of Anesthesiologists

**Fig. 2:**
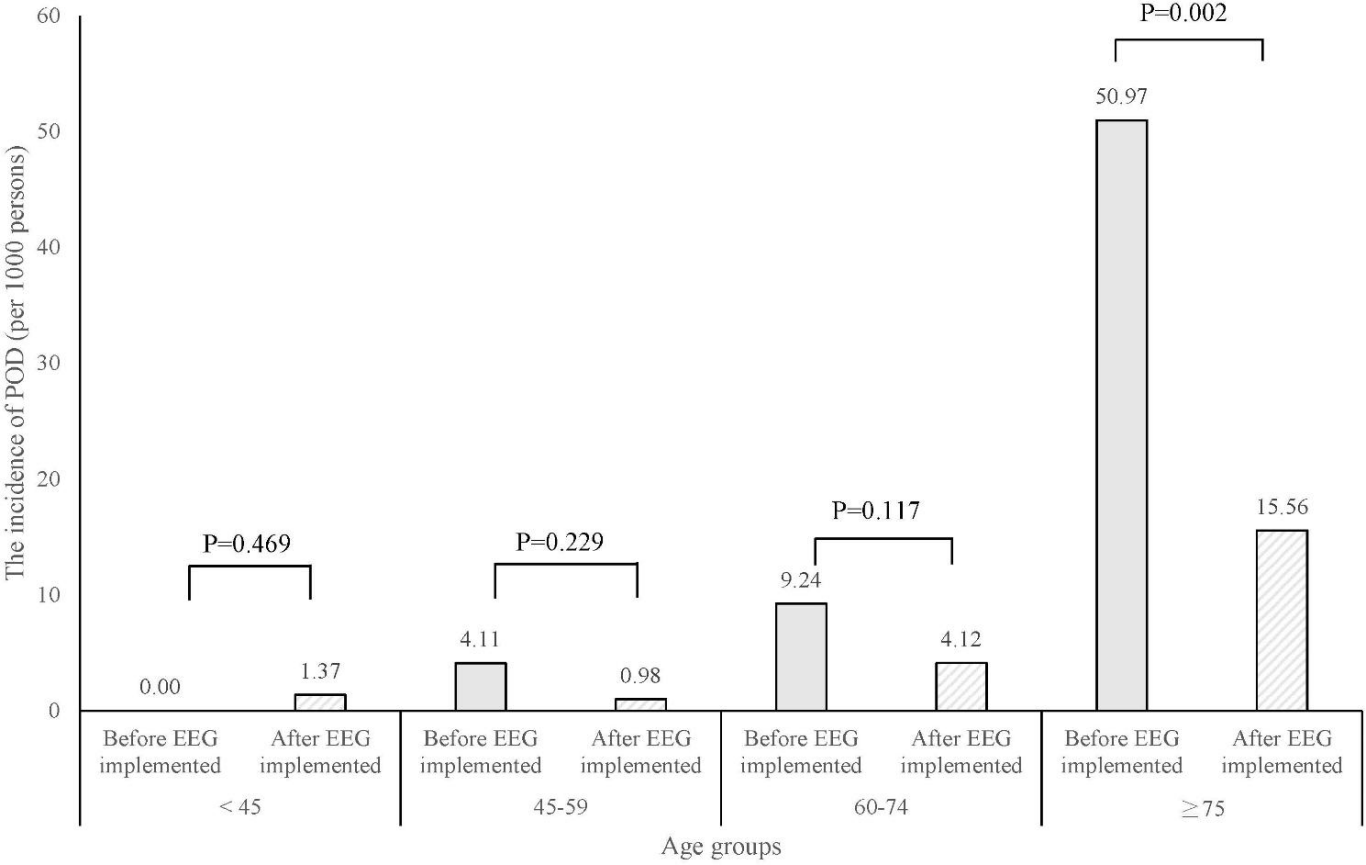
Subgroup analysis with four age categories and incidences of POD before and after intraoperative pEEG implementation

As an important confounder, age was further stratified into subgroups to compare the incidence of POD before and after pEEG implementation within each group (Fig. 2). Patients in the ≥ 75 years age group had a significantly lower incidence of POD after pEEG-guided general anesthesia implementation than before (1.56% vs. 5.1%, *P* = 0.002).

## Discussion

### Main findings

In this retrospective evaluation study, we examined the incidence change of POD after the implementation of intraoperative pEEG monitoring using the IVPCA dataset. From 2013 to 2017, we found that the incidence of POD within 3 days after surgery decreased from 1.18 to 0.41% after the implementation of intraoperative pEEG for all enrollees and from 5.1 to 1.56% for those aged ≥ 75 years.

### Strengths and limitations

The major strength of our study lies in the data retrieved from an IVPCA databank. Since all participants involved were IVPCA users, they had been adjudged to have an adequate cognitive function capable of operating the self-controlled analgesia set and making decisions independently. This ensured the baseline cognitive status of the study population. Another strength is that IVPCA users are more likely to achieve adequate pain control [26]. Targeting a pain score ≤ 3, the pain management group visited patients twice daily and aggressively modified the pain management plan. Consequently, we minimized the risk of biased results from poor cognitive function and uncontrolled pain, as either could be a strong factor contributing to delirium [7, 27, 28].

This study has some limitations. First, secular effects could not be excluded. This is inevitable when comparing postoperative outcomes before and after introducing a new monitor or concept. General anesthesia in the medical center was performed by approved anesthesiologists in line with the guideline. Standardized mean differences in demographic characteristics between the two periods show a mild difference (< 0.2). The significant association between pEEG-guided anesthesia and a lower POD rate is reconfirmed by multivariable analysis under a case-control design. Second, some factors—such as dynamic pEEG values fluctuating during surgery—are difficult to simplify into a single variable, which makes it difficult to control or adjust in the multivariable regression model. Therefore, this study can only infer the association between POD and pEEG administration but not the value maintained during surgery. Third, POD might be underdiagnosed in patients with hypoactive presentation [29, 30]. Although this was a retrospective observational study, it was built for quality management and monitoring of side effects, including delirium events, during the use of PCA. As a result, the pain management team was vigilant for any symptoms of POD. Fourth, intraoperative hemodynamic was not controlled as a covariate. However, our faculty was expected to maintain stability under general anesthesia following the same protocol, regardless of whether intraoperative pEEG monitoring was applied or not. Since the target population were intravenous patient-controlled analgesia users, patients who were likely to have poor outcomes requiring postoperative intensive care and/or prolonged sedation were not included in this study. In the second part of the study, we employed a case-control setting and adjusted for variables related to patient conditions, such as ASA classification, emergency cases, and surgery type, using a multivariable regression model. While these variables provided valuable insights, we acknowledge that they may not serve as a perfect proxy for intraoperative hemodynamic status. Finally, since the population in this study was entirely PCA users, the results may not be generalizable to patients with cognitive dysfunction or other populations. For example, minimally invasive surgeries, such as a ureteroscopy, may rarely require a PCA service.

### Interpretation

Electroencephalogram is an objective and non-invasive method for assessing neurophysiological function. A processed electroencephalogram simplifies complex brain electrical activity into a numerical index, which allows anesthesiologists to monitor anesthetic depth during surgery and minimize burst suppression [31]. Burst suppression consisting of episodes of isoelectric waves stems from deep brain inactivation, such as that caused by general anesthesia [32]. This EEG pattern was deleterious and associated with several adverse clinical outcomes, including POD [8, 33, 34]. The use of pEEG monitoring during general anesthesia has been reported to reduce anesthetic consumption and improve postanesthetic recovery [13, 35]. Furthermore, pEEG-guided anesthesia was associated with a lower incidence of POD in randomized controlled trials [36, 37]. A meta-analysis in 2018 concluded that optimized anesthesia achieved with the assistance of pEEG could reduce the incidence of POD in patients aged ≥ 60 years after noncardiac procedure [12]. Nevertheless, the ENGAGES randomized controlled trial in 2019 did not reproduce the same result. Despite reducing anesthetic exposure and duration of EEG suppression, this study showed no preventive effect on the occurrence of POD with pEEG-guided anesthetic administration [18].

Our study found that the overall incidence of POD decreased from 1.18 to 0.41% after implementing pEEG-guided anesthesia (Table 1). No event of intraoperative awareness was noted. The incidence of POD was pretty low but still within the wide range reported in previous literature [38]. One reason for the low incidence of POD is the inclusion of relatively young patients. Age is a strong factor in POD occurrence [39]. The incidences of POD in the age group ≧ 75 years are 5.1% and 1.56% before and after intraoperative pEEG, respectively. The low incidence could partly stem from the fact that patients with preexisting cognitive dysfunction were not involved as IVPCA users and adequate pain management. The trends of incidence change is presented in Fig. 1. Although no instant decrease in POD incidence in the first quarter after intraoperative pEEG-guided anesthesia administration, it reduced gradually in the following quarters. Despite well educated, clinicians reserve the flexibility of making decision according to clinical scenarios; that is, it might require some time for practitioners to recognize pEEG as a reliable tool and involve the monitor in the clinical decision-making process. Essentially, more than the pEEG results in interpreting the results and subsequent prompt management provide benefits to the patients [40].

We further analyzed the risk factors for POD in a case-control design (Table 2). After adjusting for other covariates, the odds ratio for POD would increase by 2.53 (95% CI 1.96–3.27) for every 10-year increase in age. This result is compatible with the odds ratio of 2.0 per 10 years reported by Raats et al. in 2015 [41]. While ASA classification was also mentioned as a risk factor, its association with POD was not significant in our study after adjustment for other covariates. Furthermore, patients who underwent emergency surgery had higher odds of POD (aOR 3.59, 95% CI 1.73–7.47) than those who underwent regular procedures, which is concordant with 1.5 to three times increased risk revealed in previous studies [42, 43]. Even under the setting of a case-control design, intraoperative pEEG monitoring was associated with a significantly lower risk of POD (aOR 0.33, 95% CI 0.18–0.60) after adjustment in a multivariable regression model.

Age is widely recognized as an important predisposing factor for POD [44]. Thus, we stratified patients by age groups to see if the association between intraoperative pEEG monitoring and lower incidence of POD differs in each subgroup. We found that patients of advanced age (≥ 75 years) were the only subgroup associated with a significantly lower incidence of POD (Fig. 2). One reason could be that the effect size in this subgroup was large enough to be detected. While the incidences of POD in other subgroups were less than 1%, it occurred in 50.97 per 1000 persons in the group ≥ 75 years before pEEG administration and reduced to 15.56 per 1000 persons after pEEG administration. Further, the tendency of the higher benefit of intraoperative pEEG to a more susceptible population could have been another factor. Lower brain anesthetic resistance detected by a pEEG is associated with POD [45]. As a result, anesthesia guided by pEEG might have benefitted this vulnerable population by reducing unnecessary anesthetics exposure.

## Conclusions

In conclusion, the incidence of POD decreased significantly after the implementation of intraoperative pEEG, especially in the population aged 75 years or older. In addition to using pEEG, old age and emergency surgery were independently associated with POD. This result should encourage healthcare service units to use pEEG monitoring during general anesthesia, particularly in the extremely old population. Intraoperative pEEG monitoring has been reported to be associated with numerous benefits. Even though its association with POD is controversial, pEEG could still be a competent intraoperative monitor considering its non-invasive feature and potential poor outcomes associated with POD [46]. Future prospective studies or randomized trials focusing on more extremely elderly or other susceptible populations may elucidate the benefits of intraoperative EEG further.

### Electronic supplementary material

Below is the link to the electronic supplementary material.

Supplementary Material 1

Supplementary Material 2

## Data Availability

The datasets used and analysed during the current study are available from the corresponding author on reasonable request.

## Abbreviations

POD: postoperative delirium
pEEG: processed electroencephalography
IVPCA: intravenous patient-controlled analgesia
ASA: American Society of Anesthesiologists
aOR: adjusted odds ratio
CI: confidence interval
