# Supplementary material for: Incidence change of postoperative delirium after implementation of processed electroencephalography monitoring during surgery: a retrospective evaluation study
Source: BMC Anesthesiol. 2023 Oct 4;23:330. doi: 10.1186/s12871-023-02293-9 (PMC10548752; doi:10.1186/s12871-023-02293-9)
Supplement: Supplementary file 1 — Supplementary Material 1 [file 12871_2023_2293_MOESM1_ESM.docx]

Additional file 1. Intravenous patient-controlled formulas.

| Formula | Regimen |
| --- | --- |
| I | Morphine 1 mg/ml |
| II | Morphine 0.5 mg/ml + Ketorolac 1 mg/ml |
| III | Fentanyl 20 mcg/ml |
| IV | Fentanyl 5 mcg/ml + Ketorolac 1.2 mg/ml |
| V | Morphine 0.5 mg/ml + Ketamine 1.5 mg/ml |
