# Supplementary material for: Incidence change of postoperative delirium after implementation of processed electroencephalography monitoring during surgery: a retrospective evaluation study
Source: BMC Anesthesiol. 2023 Oct 4;23:330. doi: 10.1186/s12871-023-02293-9 (PMC10548752; doi:10.1186/s12871-023-02293-9)
Supplement: Supplementary file 2 — Supplementary Material 2 [file 12871_2023_2293_MOESM2_ESM.docx]

Additional file 2. Demographic characteristic of PCA users before and after intraoperative pEEG implemented

| Variable | Before EEG implemented N (%) | After EEG implemented N (%) | *P*-value | SMD |
| --- | --- | --- | --- | --- |
|  | 3907 (100) | 3411 (100) |  |  |
| Age, mean ± SD | 57.25±15.82 | 57.17±15.48 | 0.824 | 0.050 |
| Sex |  |  |  |  |
| Male | 1783 (45.64) | 1547 (45.35) | 0.809 | 0.050 |
| Female | 2124 (54.36) | 1864 (54.65) |  |  |
| Formula |  |  |  |  |
| I (morphine) | 2014 (51.55) | 1935 (56.73) | <0.001 | 0.166 |
| II (morphine+keto) | 1792 (45.87) | 1442 (42.27) |  |  |
| III (fentanyl) | 85 (2.18) | 24 (0.70) |  |  |
| IV (fentanyl+keto) | 4 (0.10) | 8 (0.23) |  |  |
| V (morphine+ketamine) | 12 (0.31) | 2 (0.06) |  |  |
| ASA classification |  |  |  |  |
| <3 | 1468 (37.57) | 1401 (41.07) | 0.002 | 0.072 |
| ≥3 | 2439 (62.43) | 2010 (58.93) |  |  |
| Emergency | 147 (3.76) | 235 (6.89) | <0.001 | 0.140 |
| Surgery type |  |  |  |  |
| Nervous system | 29 (0.74) | 22 (0.64) | 0.026 | 0.119 |
| Endocrine system | 32 (0.82) | 27 (0.79) |  |  |
| Eye | 1 (0.03) | 1 (0.03) |  |  |
| Nose, month, and pharynx | 111 (2.84) | 88 (2.58) |  |  |
| Respiratory system | 215 (5.50) | 222 (6.51) |  |  |
| Vascular system | 22 (0.56) | 20 (0.59) |  |  |
| Hemic and lymphatic system | 4 (0.10) | 5 (0.15) |  |  |
| Digestive system | 1110 (28.41) | 914 (26.80) |  |  |
| Urinary system | 136 (3.48) | 104 (3.05) |  |  |
| Male genital organs | 28 (0.72) | 31 (0.91) |  |  |
| Female genital organs | 333 (8.52) | 362 (10.61) |  |  |
| Obstetrical procedures | 1 (0.03) | 5 (0.15) |  |  |
| Musculoskeletal system | 1548 (39.62) | 1349 (39.55) |  |  |
| Integumentary system | 259 (6.63) | 219 (6.42) |  |  |
| Others | 78 (2.00) | 42 (1.23) |  |  |

**P*-value was derived from Pearson’s chi-square test for categorical variables and Student’s t-test for continuous variables. SMD: Standardized mean difference taken absolute value.

PCA: patient-controlled analgesia; pEEG: processed electroencephalography; ASA: American Society of Anesthesiologists.

**eTable B.2. The distribution of surgery types for patients enrolled.**

| **Variable** | **POD, N (%)** | |
| --- | --- | --- |
|  | **Control group**  **7258 (100)** | **Case group**  **60 (100)** |
| **Surgery type** |  |  |
| **Nervous system** | 51 (100.00) | 0 (0.00) |
| **Endocrine system** | 58 (98.31) | 1 (1.69) |
| **Eye** | 2 (100.00) | 0 (0.00) |
| **Nose, month, and pharynx** | 199 (100.00) | 0 (0.00) |
| **Respiratory system** | 435 (99.54) | 2 (0.46) |
| **Vascular system** | 41 (97.62) | 1 (2.38) |
| **Hemic and lymphatic system** | 9 (100.00) | 0 (0.00) |
| **Digestive system** | 2007 (99.16) | 17 (0.84) |
| **Urinary system** | 238 (99.17) | 2 (0.83) |
| **Male genital organs** | 59 (100.00) | 0 (0.00) |
| **Female genital organs** | 695 (100.00) | 0 (0.00) |
| **Obstetrical procedures** | 6 (100.00) | 0 (0.00) |
| **Musculoskeletal system** | 2864 (98.86) | 33 (1.14) |
| **Integumentary system** | 476 (99.58) | 2 (0.42) |
| **Others** | 118 (98.33) | 2 (1.67) |

POD: postoperative delirium
